# Physiotherapeutic Approach Toward Restoring Upper Limb Function Post the Surgical Excision of Desmoid Fibromatosis Tumor Over the Right Upper Arm

**DOI:** 10.7759/cureus.57098

**Published:** 2024-03-28

**Authors:** Grisha R Ratnani, Vrushali Athawale, Harsh R Nathani

**Affiliations:** 1 Department of Oncology Physiotherapy, Ravi Nair Physiotherapy College, Datta Meghe Institute of Higher Education and Research, Wardha, IND

**Keywords:** rehabilitation, physiotherapy, restricted range of motion, shoulder pain, desmoid fibromatosis

## Abstract

Desmoid fibromatosis is an uncommon soft tissue tumor that is locally aggressive and can result in both pain and limited range of motion (ROM). The rehabilitation protocol is designed to promote optimal recovery and functional outcomes by following a phased approach. Initially, the focus is on managing pain and performing passive ROM exercises immediately after surgery. As the weeks progress, the exercises transition to active-assisted and then active ROM exercises. Additionally, strengthening exercises, manual therapy techniques, and functional training are incorporated to improve muscle strength, flexibility, and coordination. The goal of the rehabilitation journey is to ensure a safe return to daily activities while closely monitoring for any signs of recurrence or functional deficits. This comprehensive approach highlights the importance of collaboration between surgical teams, rehabilitation specialists, and patients in order to achieve successful outcomes.

## Introduction

Desmoid-type fibromatosis (DTF) is a rare mesenchymal neoplasm known for its invasive nature and limited capacity to spread to other areas of the body. It is a benign tumor that arises from a single clone of cells, characterized by a deep fibroblastic proliferation in the soft tissues. Desmoid tumors (DTs) exhibit infiltrative growth and tend to recur locally, but they cannot metastasize. Although they can occur in any part of the body, they are commonly found in the abdominal wall, intra-abdominal cavity, and limbs. Familial adenomatous polyposis (FAP) is associated with approximately five to ten percent of cases. DTF exhibits an inadequately defined expansion of myofibroblast-like cells accompanied by inconsistent collagen deposition, resembling the proliferative stage of wound healing. Furthermore, DTF has been linked to instances of trauma and pregnancy [1-3]. DTs possess atypical biology and demonstrate local aggressiveness while lacking the ability to metastasize, resulting in their categorization as having intermediate behavior in the WHO classification of soft tissue neoplasms. The challenging differentiation between fibromatosis and low-grade fibrosarcoma is often due to their aggressive infiltration of local structures [4].

The primary biological event involved in the development of DTs is a modification in the β-catenin pathway, leading to the nuclear buildup of β-catenin. This particular protein then interacts with transducin beta-like protein 1 (TBL1/TBLR1), forming a complex that triggers the activation of downstream genes associated with cell proliferation. In cases of sporadic desmoid fibromatosis, the majority of patients exhibit somatic mutations in catenin Beta 1 (CTNNB1), the gene responsible for encoding β-catenin. However, mutations in adenomatous polyposis coli (APC) and other genes linked to the Wnt/β-catenin pathway are rarely observed. In instances of desmoid fibromatosis associated with familial adenomatous polyposis (FAP) and Gardner syndrome, the germline mutation in APC, which is the underlying cause of FAP, is implicated in the pathogenesis of desmoid fibromatosis. This mutation leads to the production of a truncated APC protein that is incapable of binding to and facilitating the degradation of β-catenin. Consequently, the accumulation of nuclear β-catenin drives the process of cell proliferation. The occurrence of desmoid fibromatosis in individuals with APC mutations appears to be primarily linked to trauma, with up to 72% of cases in this population emerging shortly after prophylactic colectomy, either within the abdomen or in the abdominal wall [5]. MRI is considered the optimal imaging technique for accurately assessing the initial spread of DTs and tracking their progress. A core-needle biopsy guided by imaging is necessary to establish a formal diagnosis of DTs [6].

## Case presentation

Patient information

**Figure 1:**
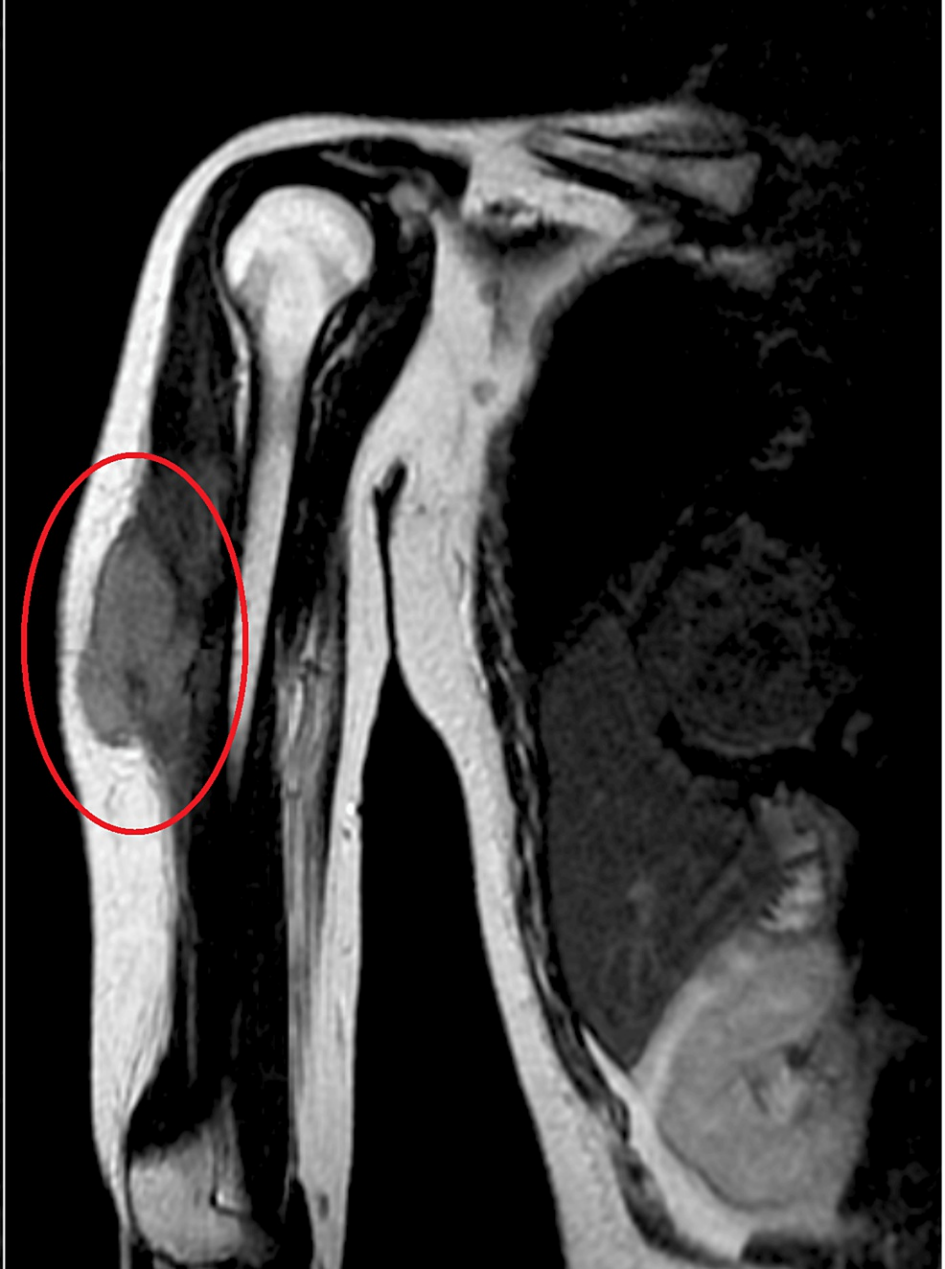
MRI of the right arm The red circle region shows a well-defined mass lesion involving the distal deltoid muscle and the adjoining lateral head of the triceps muscles.

A 31-year-old female patient arrived at the hospital with swelling on her right upper arm laterally that had been present for five months. The swelling had gradually developed and was progressively worsening. Additionally, over the past two months, she had been experiencing a dull aching pain that extended to her forearm. The patient disclosed a history of trauma eight months ago when she fell from her two-wheeler and collided with a brick on her right upper arm. Following the accident, she noticed a bruise but did not seek medical attention. Upon examination, a fixed mass measuring approximately 5×4 cm was discovered on the lateral aspect of her right upper arm. The surface of the swelling was smooth, firm in consistency, with unclear boundaries, tender (grade 3), and immobile. An MRI scan revealed a large, well-defined mass lesion involving the distal deltoid muscle and adjoining the lateral head of the triceps muscles, which exhibited T2 hyperintensity and T1 iso-intensity. Additionally, a well-defined multilobulated lesion with T2 diffuse hyperintensity was observed in the latissimus dorsi muscle in the right lateral upper chest wall, raising suspicion of a low-flow venous vascular malformation or neoplasm, necessitating further biopsy. The biopsy confirmed the presence of desmoid fibromatosis, characterized by a cellular lesion composed of uniform, long, slender, spindle-shaped fibroblasts arranged in bands and bundles, separated by varying amounts of fibrous stroma. A surgical intervention was performed on August 24, 2023, to excise the tumor. However, after the surgery, the patient persisted in complaints of dull aching pain around the excision site and restricted ROM in the right shoulder joint. Consequently, the patient was referred to the physiotherapy department for further management. Figure 1 shows the MRI of the right shoulder with the lesion marked with a red circle.

Clinical evaluation

During the clinical evaluation, the patient was assessed while lying in a supine position. The patient reported a dull aching pain rating of 8 out of 10 on the visual analog scale, specifically on the lateral aspect of the right upper arm during activity. The pain was exacerbated during activities such as eating and lifting objects with the affected arm but was relieved with rest. The patient is 152 cm tall, weighs 48kg, has an ectomorphic build, and shows no signs of pallor, icterus, clubbing, or cyanosis. Upon palpation, there was mild swelling and grade 2 tenderness present on the lateral aspect of the right arm. Muscle wasting was also observed in shoulder abductors and elbow extensors. The ROM and muscle strength assessments are detailed in Table 1, indicating a noticeable restriction in movement and a significant reduction in strength.

**Table 1:** Physiotherapeutic assessment of affected (right) upper limb. "Grade 3-": (Fair-) Some but not complete ROM against gravity. "Grade 3": (Fair) (50%) Complete ROM against gravity. "Grade 2+": (Poor+) Initiates motion against gravity. ROM: range of motion

Joint	Movement	Quality of movement	Range of motion	End-feel	Strength of the muscle responsible for movement (modified Oxford scale)
Shoulder	Flexion	Painful and incomplete	0-50°	Empty	Grade 3-
	Extension	Painful and incomplete	0-15°	Empty	Grade 3-
	Abduction	Painful and incomplete	0-45°	Empty	Grade 2+
	Internal rotation	Painful and incomplete	0-30°	Empty	Grade 3-
	External rotation	Painful and incomplete	0-40°	Empty	Grade 3-
Elbow	Flexion	Painful and incomplete	0-120°	Empty	Grade 3

Management

Surgical Management

The patient underwent a surgical procedure known as wide local excision to remove a solid mass measuring approximately 8 x 6 cm. This mass was located between the muscles in the lateral compartment of her right arm. The surgery involved making an elliptical incision over the affected area, ensuring that enough healthy tissue was included. This incision encompassed the site of a previous biopsy and the surrounding skin. Flaps of tissue were raised to expose the bone (humerus) and a partial removal of the deltoid and triceps muscles was necessary. A few adjacent fibers of the lateralmost deltoid were excised. The fascia, a layer of connective tissue, was identified and the tumor mass was carefully separated from the surrounding structures. The tumor, along with a 2-3 cm margin of healthy tissue, including a portion of the periosteum of the humerus over its one-third midshaft, was completely excised. Throughout the procedure, special attention was given to preserving the radial nerve. Hemostasis, or the control of bleeding, was confirmed and Ligaclips were applied to the margins of the tumor bed to secure it. A drain was placed beneath the fascia to prevent fluid accumulation, and the skin was sutured. The surgical management was successful, and on the third day after the operation, the drain was removed and the patient was discharged from the hospital with medications for pain (tablet Zerodol-Sp).

Physiotherapeutic Intervention

Physiotherapeutic intervention is crucial in maximizing the patient's recovery and functional results post-surgical removal of desmoid fibromatosis in the upper right arm. A customized rehabilitation regimen was devised with the goal of managing pain, reinstating ROM, enhancing muscle strength, and boosting overall functional ability. Rehabilitation began on the third postoperative day. The treatment plan followed a step-by-step process, starting with gentle passive and active-assisted exercises to prevent stiffness and encourage joint mobility, and progressing to scar care, strength-building exercises, and functional training to restore functional autonomy. The intervention was planned for six weeks. Table 2 summarizes the detailed physiotherapeutic strategies involved in managing the patient.

**Table 2:** Detailed physiotherapy protocol administered for six weeks. TENS: transcutaneous electrical nerve stimulation; ROM: range of motion; PNF: proprioceptive neuromuscular facilitation

Phase of rehabilitation	Goals	Rehabilitation strategies
Immediate post-rehabilitation phase (days 1-7)	Patient education and counseling	Comprehensive education regarding her condition, rehabilitation objectives, and treatment strategy, emphasizing the significance of adhering to prescribed exercises and pain management techniques
		Modification of her activities, maintaining a nutritious diet, and staying properly hydrated to facilitate optimal recovery
		Explanations of warning signs of infection, such as inflammation and fluid discharge
	To manage pain	Cryotherapy for 10 minutes thrice a day
		TENS for 10 minutes
	To control edema	Keeping the affected limb in elevation with a cushion underneath
	To prevent joint stiffness and maintain joint mobility	Pendular exercises for the shoulder for 5-10 minutes thrice a day
Early rehabilitation phase (days 8-21)	To improve the ROM of the shoulder	Active assisted ROM exercises for shoulder flexion, abduction, and external rotation
		Finger ladder exercises, one set of 10 repetitions.
		Stretching of pectorals, trapezius, and tricep
	To improve the strength of the shoulder, elbow, and wrist musculature	Initiating with isometric exercises for shoulder flexors, abductors, rotators, elbow flexors, and wrist muscles. Strengthening exercise for scapular retractors and elevators
		Grip strengthening exercises using a gel ball
	To improve mobility	Scapular mobilization
	Scar tissue management	Scar tissue massage
Intermediate rehabilitation phase (weeks 3-6)	Progressing strength training	Resistance training exercises using theraband and dumbbell, gradually increasing the intensity
	To improve ROM	Mobilization with movement to improve abduction and external rotation (10 repetitions)
	Functional training	D1 and D2 flexion-extension patterns of PNF using therabands two sets of 10 repetitions

Follow-up and outcome measures

The assessment was conducted following each stage of the rehabilitation process. Subsequent to a six-week period of focused rehabilitation, the affected limb attained a functional range of motion, and notable enhancements in strength and movement quality were observed, as depicted in Table 3.

**Table 3:** Physiotherapy assessment of range of motion, quality of movement, and muscle strength after six weeks of rehabilitation. "Grade 4": (Good (75%)) Complete ROM against gravity with some (moderate) resistance. "Grade 3+": (Fair+) Complete ROM against gravity with minimal resistance ROM: range of motion

Joint	Movement	Quality of movement	Range of motion	Strength of the muscle responsible for movement (modified Oxford scale)
Shoulder	Flexion	Painless and complete	0-165°	Grade 4
	Extension	Painless and complete	0-55°	Grade 4
	Abduction	Painless and complete	0-160°	Grade 3+
	Internal rotation	Painless and complete	0-65°	Grade 4
	External rotation	Painless and complete	0-75°	Grade 4
Elbow	Flexion	Painless and complete	0-130°	Grade 4

Table 4 shows the outcome measures utilized for the assessment. The Visual Analog Scale (VAS) has been shown to possess a strong level of dependability in evaluating acute pain [7]. The Shoulder Pain and Disability Index (SPADI) was developed to evaluate the extent of shoulder pain and disability in individuals undergoing treatment in an outpatient setting [8]. The Disability of the Arm, Shoulder, and Hand (DASH) Questionnaire is a self-administered instrument intended to evaluate disability and symptoms in the upper extremities within a particular body region [9]. 

**Table 4:** Weekly assessment of outcome measures. VAS: Visual Analogue Scale; DASH: Disabilities of the Arm, Shoulder, and Hand Questionnaire; SPADI: Shoulder Pain and Disability Index

Outcomes	Week 1	Week 2-3	Week 6
VAS	8/10	5/10	1/10
DASH	46%	28%	12%
SPADI	38%	24%	10%

## Discussion

Desmoid fibromatosis, despite being histologically benign, poses distinctive difficulties in terms of treatment and recovery. The presented case report underscores the significance of thorough rehabilitation post the surgical removal of desmoid fibromatosis in the upper right arm to enhance functional results and the overall well-being of those impacted. The discourse pertaining to this case explores various critical aspects.

Patients diagnosed with DTs often face a significant symptom burden. Studies have shown that as many as 63% of patients with DTs suffer from chronic pain, resulting in sleep disturbances in 73% of cases, irritability in 46% of cases, and anxiety or depression in 15% of cases. Commonly reported symptoms include pain, restricted function and mobility, fatigue, muscle weakness, and swelling near the tumor site. As a result, the overall quality of life for individuals with DTs tends to be lower compared to healthy individuals [10]. DTs have a tendency to infiltrate neighboring organs, spread through fascial planes, exert pressure on blood vessels and nerves, erode bones, or obstruct organs like the bowel. The involvement of muscles, nerves, and vessels can lead to severe symptoms, such as pain, limited mobility, or deformity. For instance, tumors that affect the extremities can restrict joint movements, resulting in difficulty in moving the upper and lower extremities while walking [11,12].

The aggressive local behavior and high recurrence rates of this lesion compel surgeons to make difficult decisions and implement complex treatments. Options include surgical resection, radiotherapy, hormonal therapy (tamoxifen or toremifene), and the use of non-steroidal anti-inflammatory drugs. The principal determinant of outcome remains a radical resection with free margins, making surgery the primary treatment [13]. A significant proportion of cancer survivors will encounter difficulties with physical and cognitive function as a consequence of cancer treatments and associated adverse effects. These functional limitations can impede their involvement in social and occupational roles, leading to a decrease in their quality of life. Nonetheless, interventions such as rehabilitation and exercise have been proven to alleviate the adverse effects of treatment-related symptoms and enhance the functional capacity of affected patients [14].

Functional impairments following desmoid fibromatosis in the deltoid and tricep regions present a variety of obstacles, such as limited shoulder movement in abduction, flexion, and external rotation, as well as reduced muscle strength and endurance. These challenges often result in difficulties performing tasks that require upper limb function, like lifting, reaching, and pushing, impacting one's autonomy and overall well-being. The persistent pain and discomfort in the affected area worsen these functional limitations, making it harder to participate in daily activities and recreational pursuits. Rehabilitation methods, such as specific exercises, manual therapy, and functional training, play a crucial role in alleviating these limitations and facilitating recovery. By receiving collaborative care and personalized rehabilitation plans, individuals can restore their functional autonomy and resume essential daily activities.

Physiotherapy in the field of oncology is intricately linked to the methods employed for the management of tumors. The process of rehabilitation primarily revolves around engaging in physical activities that target the musculoskeletal system while also having a profound impact on the emotional well-being of individuals. The incorporation of physical activity plays a pivotal role in enhancing both the physical and psychological welfare of those undergoing surgical treatment [15].

## Conclusions

The recovery process following the surgical removal of desmoid fibromatosis in the upper right arm is crucial for restoring the optimal function of the arm and improving the mobility of patients. By implementing a personalized rehabilitation plan that focuses on specific goals such as improving ROM, muscle strength, pain management, and overall functionality, significant progress can be achieved in enhancing patient outcomes. The collaboration between surgical teams, rehabilitation specialists, and patients is vital in ensuring successful rehabilitation results. By closely monitoring the progress and making necessary adjustments to the rehabilitation program, long-term independence and a reduced risk of recurrence or complications can be achieved. Ultimately, this comprehensive approach highlights the significance of patient-centered care and evidence-based rehabilitation strategies in facilitating a seamless transition from surgery to functional recovery. It empowers individuals to regain control over their daily activities and lead fulfilling lives after surgery.
